# Exploring residents’ perceptions of competency-based medical education across Canada: A national survey study

**DOI:** 10.12688/mep.19247.1

**Published:** 2024-01-08

**Authors:** Heather Braund, Vivesh Patel, Nancy Dalgarno, Steve Mann

**Affiliations:** 1 Professional Development & Educational Scholarship, Queen's University, Kingston, Ontario, K7L 1B9,, Canada; 2 Faculty of Health Sciences, Queen's University, Kingston, Ontario, K7L 2Y1, Canada; 3 Department of Surgery, Queen's University, Kingston, Ontario, K7L 2V7, Canada

**Keywords:** Competency-based medical education, resident experiences, resident perspectives, survey

## Abstract

**Background:** As competency-based medical education (CBME) is implemented across Canada, little is known about residents’ perceptions of this model. This study examined how Canadian residents understand CBME and their lived experiences with implementation.

**Methods:** We administered a survey in 2018 with Likert-type and open-ended questions to 375 residents across Canada, of whom 270 were from traditional programs (“pre-CBME”) and 105 were in a CBME program. We used the Mann-Whitney test to examine differences across samples, and analyzed qualitative data thematically.

**Results:** Three themes were identified across both groups: program outcome concerns, changes, and emotional responses. In relation to program concerns, both groups were concerned about the administrative burden, challenges with the assessment process, and feedback quality. Only pre-CBME residents were concerned about faculty engagement and buy-in. In terms of changes, both groups discussed a more formalized assessment process with mixed reactions. Residents in the pre-CBME sample reported greater concerns for faculty time constraints, assessment completion, and quality of learning experiences, whilst those in CBME programs reported being more proactive in their learning and greater selfreflection. Residents expressed strong emotional narrative responses including greater stress and frustration in a CBME environment.

**Conclusion:** Findings demonstrate that residents have mixed feelings and experiences regarding CBME. Their positive experiences align with the aim of developing more self-directed learners. However, the concerns suggest the need to address specific shortcomings to increase buy-in, while the emotional responses associated with CBME may require a cultural shift within residency programs to guard against burnout.

## Introduction

Canadian postgraduate medical education (PGME) programs are transitioning from a time-based model to competency-based medical education (CBME) ^1^. The Royal College of Physicians and Surgeons of Canada (RCPSC) introduced CBME with the 2017 cohort of residents at one Canadian institution. Since then, Canadian PGME programs have been transitioning to the RCPSC competency-based model, competence by design (CBD) ^1^.

CBME is supported by four tenets including a focus on outcomes, an emphasis on abilities, a de-emphasis of time-based training, and the promotion of learner-centredness. Another benefit of CBME is the requirement for frequent, utilitarian assessment ^2^. This increased assessment is intended to promote learner-centred teaching that could help residents achieve the required competencies ^2^. These benefits informed CBME implementation, with the goal of standardizing and improving residency training across Canada, and fostering self-reflection and lifelong learning skills. Implementing this significant change has required much scholarship and collaboration ^2– 5^. However, although educational scholars have written extensively about CBME, there remains a gap in the literature examining resident perspectives ^6, 7^. CBME literature has focused on examining the impact of educational interventions on residents’ understanding of the process and purpose ^8^, or utilizing qualitative methodology ^9^. Additional areas of focus include understanding residents’ perceptions specific to assessment ^10, 11^. The literature also focuses on resident perspectives within specific programs including internal medicine ^10, 11^, obstetrics and gynecology ^12^, and anaesthesia ^13^ rather than national data across specialities.

Research has shown that residents anticipate improved assessment, feedback, and flexibility to pursue self-identified educational needs, but are concerned regarding logistical challenges and administrative burden ^9^. These perspectives are important, because residents are key stakeholders as learners, mentors, and assessors. Gaining increased insight into residents’ perspectives will support an environment that makes learning more efficient and meaningful. Furthermore, residents’ perspectives provide insight into the implementation of CBME programs and many of the daily intricacies of CBME that might otherwise go unnoticed. Given the rich knowledge that can be discovered from residents’ perspectives, they should be targeted to improve CBME implementation and help ensure the goals are being met.

This exploratory study examined how Canadian residents conceptualize CBME with a goal to gain insight and add to the discourse around CBME. This study may aid the RCPSC and program directors as they implement CBME and make evidence-informed decisions regarding their programs. Sharing the resident voice may also enhance the resident experience, improve buy-in, and ultimately cultivate more competent physicians.

## Methods

This study examined Canadian residents’ perceptions of CBME before and after implementation through a survey design.

### Ethical statement

This study received ethics approval through the institutional Health Sciences Research Ethics Board (File number: 6020800). Participants provided written informed consent for participation in the study and the use and publication of their data. If they desired, they were entered into a draw for compensation which included a draw for one of two Apple Watches.

### Setting and participants

Scholars at a mid-sized university in Eastern Ontario recruited residents in time-based and CBME RCPSC training programs. We distributed the online survey examining residents’ perceptions of CBME from July 2018 through December 2018 to trainees in English-speaking Canadian residency programs, by means of an electronic notice sent through institutional list servers. A total of 10 Canadian institutions circulated the survey. However, given that the survey was distributed by institutions, the sampling frame is unknown as each institution used different listservs and varied numbers of reminders.

### Data collection and tools

This study is a continuation of a project that started with individual interviews; those lived experiences informed survey development for this study ^9^.

We collected data through a single-phase design consisting of a survey which included closed-ended items (Likert) and an open-ended question. The survey branched to questions according to whether participants indicated they were in a CBME program. The questions contained the same premise but were re-worded to make sense for participant context, either ‘pre-CBME’ or ‘in CBME’.

A total of 12 Likert items were divided into two categories, 1) assessment and feedback, and 2) learning experiences. Participants rated their agreement using a six-point scale (1=Strongly disagree, 2-Disagree, 3=Neither agree nor disagree, 4=Agree, 5=Strongly agree, and 6=Prefer not to answer). After the Likert items, participants received an open-ended question asking how CBME might affect their residency experience (pre-CBME) or how CBME has affected their residency experience (in CBME). The final question used a six-point Likert scale where residents rated their overall satisfaction with their current educational training (1=Very dissatisfied, 2=Dissatisfied, 3=Neither satisfied nor dissatisfied, 4=Satisfied, 5= Very satisfied, and 6=Prefer not to answer.) No demographic data were collected as a means of protecting resident identity ^14^ given that some residency programs in Canada are small. We piloted the survey between April 2018 and May 2018 and evaluated the items for their construct validity ^15^. The survey was completed by 13 respondents external to the study who provided feedback on item wording, flow, completion time, and item interpretation. These respondents were purposefully selected based upon their expertise in survey design, knowledge of CBME, and interest in medical education. This process resulted in small refinements (e.g., clarifying terminology and rewording). We used Cronbach’s alpha to evaluate survey reliability ^16^.

### Data analysis

**Quantitative:** Data were de-identified and participants were assigned a study ID number for the analysis. Quantitative data were uploaded into SPSS Statistical Package (version 25) for analyses. Our sample violated normality assumptions; hence we used the Mann-Whitney U to examine differences across samples, and significance was set as .05. 

**Qualitative:** All qualitative data were analyzed thematically using NVivo (version 12). The smallest level of analysis was a code; we grouped similar codes together into categories, and grouped similar categories together to form themes ^17^. Two researchers coded 25% of the data independently and then met to discuss their coding with an inter-coder reliability of 97%. The discussion resulted in a consensus-built codebook used for the coding. We reached data saturation, with no new findings emerging from the last 20 responses. Once preliminary themes were determined the research team met to discuss the findings and ensure accurate data representation.

### Researcher reflexivity

The lead author is an assistant professor and orthopaedic surgeon with a Master’s in Medical Education. The two researchers are mixed methodologists with extensive experience conducting educational scholarship. A medical student who was learning how to conduct research was also involved. The researcher and medical student conducted the coding and statistical analysis before discussing all results with the team. The researchers engaged in a reflexive process including the development of a coding diary to identify common patterns, possible biases, and document memos. This process ensured that the interpretation of data was in alignment with participants’ perspectives. The activities promoting reflexivity and the coding process described above is in alignment with recommended guidelines for establishing inter-coder reliability.

## Results

A total of 375 residents completed the survey, of whom 270 were pre-CBME (72% of respondents) and 105 were enrolled in a CBME program (28% of respondents). A Cronbach’s alpha score of .691 indicated that the Likert-items were approaching acceptable reliability ^18^. 86% of respondents provided narrative responses.

### Effect of CBME on assessment and feedback

An overview of the differences between groups is available in Table 1. Residents in the pre-CBME group had greater concerns that faculty time constraints would affect assessment completion ( *Mean* = 4.13) than CBME residents ( *Mean =* 3.91), *U* = 11663.50, *z* = -2.88, *p* = .004, *r* = -0.14. Similarly, pre-CBME residents ( *Mean* = 3.28) were skeptical about the level of faculty CBME knowledge and believed that faculty were less likely to have the required knowledge to complete assessments compared to CBME residents ( *Mean* = 3.74), *U* = 1698.50, *z* = -3.84, *p* = .000, *r* = -0.20.

**Table 1.** Differences between groups for effect of competency-based medical education (CBME) on assessment and feedback.

	Faculty time constraints will limit the extent to which they can complete the assessment tools and provide feedback.	The need for frequent assessments from faculty will make it difficult for residents to complete CBME assessment tools.	Faculty in my department have the knowledge required to complete quality assessments using the CBME model.	Faculty in my department will be able to make valid and reliable decisions about residents' competence using the CBME model.	Definitions of competence, competencies, EPAs, and milestones will be consistent among assessors and competency committees.	Assessment in the CBME model will result in more valuable feedback from faculty.	Assessment in the CBME model will positively impact faculty’s ability to coach residents.
*N* Pre- CBME	270	270	270	270	270	270	270
*N* CBME	105	105	105	105	105	105	105
Means Pre-CBME	4.13	3.96	3.28	3.51	2.83	3.53	3.51
Means CBME	3.91	3.68	3.74	3.6	3.14	3.5	3.47
Z	-2.88	-2.24	-3.84	-.76	-2.46	-.96	-.68
P Value	**.004**	**.025**	**.000**	.445	**.014**	.338	.497

### Effect of CBME on resident learning experiences

An overview of the differences is available in Table 2. The only significant difference found was that pre-CBME residents ( *Mean* = 3.46) agreed that faculty would spend greater time on administering CBME than on learning experiences when compared to CBME residents ( *Mean* = 2.91), *U* =9705.50, *z* = -1.095, *p* = .000, *r* = -0.26. Residents in both groups reported high satisfaction, with pre-CBME residents having higher satisfaction ( *Mean* = 4.26) when compared to those in CBME programs ( *Mean* = 4.19).

**Table 2.** Differences between groups for effect of competency-based medical education (CBME) on resident learning experiences.

	In the CBME model, faculty may spend more time on administering a competency-based programme than on ensuring the quality of the learning experiences.	The CBME model will accommodate individual learning needs and variability in the pace of learning.	The CBME model will encourage resident self- reflection.	The CBME model will encourage residents to engage in self-directed learning.	In the CBME model, once residents identify their learning needs, they will pursue learning opportunities to achieve competence in those areas.	Please rate your overall satisfaction with the quality of education you receive in your current program.
*N* Pre- CBME	270	270	270	270	270	270
*N* CBME	105	105	105	105	105	105
Means Pre-CBME	3.46	3.46	3.66	3.59	3.60	4.26
Means CBME	2.91	3.35	3.79	3.69	3.69	4.19
Z	-4.96	-1.09	-1.18	-1.01	-.79	-.97
P Value	**.000**	.273	.239	.314	.432	.332

### Qualitative findings

Three themes were identified: 1) Program outcome concerns, 2) Changes, and 3) Emotional responses. Whilst these themes were evident across groups, the frequency with which they were mentioned differed. Table 3– Table 5 provide supporting quotations for each theme. Additional quotations can be found in Table 6.

**Table 3.** Supporting quotes for Theme 1: Program outcome concerns.

Sub-theme	Supporting Quote
Administrative burden	“ *Incessant burden of paperwork*” (P269, Pre-CBME) “ *I suspect it will be more busy work whereas as a member of a smaller program I think I get enough* * supervision feedback and personalized attention*” (P274, Pre-CBME) “ *Extra stressor in terms of administrative burden to have assessments done for daily tasks*” (P98, In CBME).
Assessment challenges	“ *Difficulty in getting staff to do the evaluations during the workday*” (P105, Pre-CBME) “ *More paperwork but more observed encounters too which are appreciated*” (P18, In CBME)
Quality of feedback	“ *At times feedback is quite generic, but no different than prior to CBME*” (P40, In CBME) “ *I think it will be very beneficial in obtaining and seeking effective feedback*.” (P221, Pre-CBME).
Faculty engagement and buy-in	“ *Staff in my program already don't do Midterm assessments and end of rotation assessments. There is no* * way they are going to do competency forms*” (P175, Pre-CBME). “ *I find it stressful to try to get feedback on off-service rotations because the staff there don't really care*” (P138, In CBME).

**Table 4.** Supporting quotes for Theme 2: changes.

Sub-theme	Supporting Quote
Formalized assessment process	“ *More formal assessments*” (P174, In CBME) “ *I anticipate it will formalize evaluation of the learning objectives already in place, to document in greater detail whether* * residents are in fact meeting all of their rotation objectives*” (P242, Pre-CBME) “ *It is burdensome to approach faculty as there is a negative outlook on assessment as it is a frustrating process right* * now*” (P64, in CBME)
Teaching and learning process	“ *There are certain objectives that I need to reach according to the CBME model, but staff have not changed their teaching * *style*” (P126, In CBME) “ *Less time receiving teaching from attendings given need for assessments*” (P174, Pre-CBME). “ *Staff will be more focused on filling out forms than on patient specific teaching*” (P2, Pre-CBME) “ *Changing the way that I instruct learners as a senior resident*” (P15, Pre-CBME). “ *I feel it has encouraged me to be a self-directed learner*” (P136, In CBME) “ *Being able to concentrate on the things I really am lacking in and minimize time spent on things I know very well*” (P213, Pre-CBME) “ *I feel CBME is overall positive because it allows a resident to progress at the rate they feel comfortable*” (P234, Pre-CBME)

**Table 5.** Supporting quotes for Theme 3: emotional responses.

Sub-theme	Supporting Quote
Negative feelings	“ *I think that it would be more concise and useful, but it will also require more time and will probably be more stressful*” (P18, In CBME) *“It is estimated that our minds can accommodate ~7 items at a time. Between consults, paperwork, and ward issues* * we often become stressed. Adding a requirement to fill out at least 3 assessments a day (as estimated by my surgical* * PD last week) will increase distraction and stress, hopefully not limiting patient care”* (P95, Pre-CBME) *CBME has put a lot of stress on me in my residency. Faculty do not fill out assessments. I barely ever get them back*” (P7, In CBME). “ *I feel an extra stress on shift of completing EPAs as opposed to focussing on patients and flow*” (P35, In CBME) “ *Slightly more stressful as school transitions to CBME model and overcomes growing pains*” (P65, In CBME) *“I find this constant feedback to be emotionally taxing. To elaborate, I find preceptors feel the need to always * *say something good you did and something bad you did even if both were not necessary. As a result, I feel * *like eval[uation]s have gotten way more nit-picky and consequently anxiety inducing”* (P101, In CBME). “ *Not yet sure. I worry about a lack of flexibility*” (P178, Pre-CBME) “ *I think it will encourage feedback more often. However, I worry about how difficult it will be to encourage staff to* * actually participate and be engaged in more frequent feedback*” (P190, Pre-CBME).

**Table 6.** Additional quotations for each theme.

Theme	Subtheme	Sample Codes	Sample Quotations
1. Program outcome concerns	a) Administrative burden b) Assessment challenges c) Quality of feedback d) Faculty engagement and buy-in	-Greater administrative burden Assessment completion Quality Feedback Will Be Challenging Hesitant to be constructive Staff engagement	“Higher administrative burden on residents to pursue and complete evaluations.” ( *P99, Pre-CBME*) “More time spent doing paperwork” ( *P132, In CBME*) “I fear there will be a significant gap in supervisor skill, and willingness to dedicate the time needed, to assess milestones/ EPAs when CBME is first implemented.” ( *P245, Pre-CBME*) “I am in R1 in a program transitioning to CBD for my year. It is a learning curve for staff in the Emergency department as they don't like having to actually observe me taking histories or doing physicals.” (P45, In CBME) “But attending physicians are often very busy, measures need to be in place so that the quality of feedback is not compromised.” ( *P122, Pre-CBME*) “Assessors are hesitant to give constructive feedback” ( *P59, In * *CBME)* “However, I worry about how difficult it will be to encourage staff to actually participate and be engaged in more frequent feedback.” ( *P188, Pre-CBME*) “Now, a year later, that has somewhat passed, and it is more of a hassle to track down staff / convince them to fill EPAs that it is a benefit.” ( *P105, In CBME*)
2. Changes	a) Formalized assessment process b) Teaching and learning process	More documentation Tailored teaching No difference in teaching Decreased learning	“I anticipate I will spend more time completing documentation of clinical experiences/EPAs” (P25, Pre-CBME) “There will be more time spent documenting tasks” (P114, Pre-CBME) “I think I will get more tailored teaching to my level of understanding” (P177, Pre-CBME) “While it was fresh in the first few months of introduction, staff would seek out opportunities to involve me in more complex cases (that aligned with complex EPAs). Now, a year later, that has somewhat passed, and it is more of a hassle to track down staff / convince them to fill EPAs that it is a benefit. It seems, aside from having forms filled, the teaching is not much different.” (P105, *In CBME*) “I find more of my time is spent on the logistics of CBME (chasing down evaluations, keeping track of my progress, competence reports etc.) rather than actually learning the content behind EPAs.” (P4, In CBME)
3. Emotional responses	a) Negative feelings	More stress Worried Stressed	“Added stress of ensuring frequent assessments by staff in a busy clinic or unit” (P115, Pre-CBME) “I am personally worried about staff not being willing or not having time to the CBME EPAs and evaluations which could potentially blow back on residents and making it hard for them to complete EPAs not from lack of trying, but from time constraints or other limitations outside of our control.” (P55, Pre-CBME) “Trying to obtain the required competencies, explain CBME milestones/EPAs to preceptors, arrange direct observation, and have preceptors complete the required forms has been by far the most stressful part of residency so far. It has taken away from my learning experiences, as I target my patient encounters towards seeing the patients I need to check-off from my list rather than those that are interesting, and it deters me from seeing challenging patients that I know I would receive a poor evaluation for” (P77, In CBME)

### Theme 1: Program outcome concerns

Many residents were concerned about administrative burden, assessment challenges, feedback quality, faculty engagement, and buy-in ( Table 3). However, residents in the pre-CBME group were more concerned about increased paperwork and administrative burdens. Residents experiencing CBME identified the administrative burden as a stressor.

 Concerns were raised about the assessment process, such as assessment completion during clinical time. Whilst some CBME residents reported challenges with assessment completion and direct observation, others described increased direct observation as a benefit. Another concern was how feedback quality would be impacted, as some pre-CBME residents anticipated better feedback whereas some CBME residents reported no change in their feedback.

Residents anticipated challenges due to lack of engagement and buy-in from staff. Pre-CBME residents discussed issues with assessment completion before CBME which were expected to be an ongoing challenge. Residents in CBME expressed lack of engagement and buy-in to a lesser extent. Some residents reported additional stress from CBME implementation.

### Theme 2: Changes

Residents identified processes that may change or had changed including formalized assessment, teaching, and learning ( Table 4). The pre-CBME residents focused on how the assessment process would become more formalized through direct observation and increased documentation. A few CBME residents reported that the increased documentation and requirements for assessment caused frustration.

Some CBME residents discussed how teaching had not changed following CBME implementation. Pre-CBME residents also discussed anticipated changes to teaching, as many were concerned about receiving less teaching due to assessment processes. Some residents identified the need to change their own teaching styles.

CBME residents reported that they were better able to direct their learning following implementation. Some pre-CBME residents indicated that they were excited for more tailored learning experiences and to focus on specific areas of need. They also anticipated a more individualized learning pace. There were clearly positive and negative implications for assessment, teaching, and learning in a CBME environment.

### Theme 3: Emotional responses

A theme related to emotional responses due to CBME ( Table 5) was identified. Generally, these emotions were negative and included stress, uncertainty, anxiety, and worry. Some pre-CBME residents were anticipating increased stress. The experience of added stress was also reported by CBME residents. A few residents expressed how the added stress could negatively impact patient care given that they were focused on completing entrustable professional activities (EPAs), rather than on patients and clinical flow. The amount of stress experienced varied among residents; some reported a high level of stress and anxiety, and others did not mention stress.

 Other feelings included uncertainty as some residents were unsure how CBME would impact them. Sometimes the worry was in relation to faculty buy-in, engagement, and assessment completion. Despite not being asked about their emotions, residents shared how they were feeling about CBME.

## Discussion

Canadian residents have mixed feelings and experiences about CBME. Our findings suggest that apprehensions held by pre-CBME residents were less concerning to CBME trainees. Statements suggesting difficulties with time constraints and engagement related to assessment completion had significantly lower agreement amongst CBME residents. However, while CBME residents were significantly less concerned about variability in defining competence, the mean score of 3.14/5 (compared to 2.83/5 for pre-CBME residents) indicates that there is still work required to instill confidence in trainees regarding the ability of the CBME system to provide clear definitions and measurable metrics of competence. Residents shared tensions from assessment processes which is understandable given that within the CBME model we remain situated in an accountability paradigm and are using assessments for high-stakes decisions. However, future work should focus on identifying tensions and documenting how residents and faculty are navigating them ^19^. Further, we need to emphasize learning and de-emphasize measurement within CBME ^20^.

Self-reflection and self-directed learning received strong agreement scores across groups, suggesting that this is a major benefit of CBME. Conversely, CBME residents were less optimistic that individual learning needs and variability in learning pace were being accommodated, which suggests that CBME still exists within the logistical demands and framework of rotation schedules, call coverage, and patient care. Reassuringly, both groups reported high satisfaction with their training.

86% of respondents provided narrative responses, yielding rich qualitative data. These responses confirmed the quantitative findings: concerns regarding the logistical and administrative aspects of CBME are still present post-implementation, and the time demands of assessment and documentation inherent in CBME, although perhaps not quite as onerous for the pre-CBME group, remain significant. Residents are aware of the magnitude of the change required, requesting adaptation of assessment and teaching strategies.

The negative emotional responses expressed by participants were surprising and concerning, although one study highlighted emotional responses of trainees during CBME implementation including frustration due to assessment processes ^21^. Additionally, the departmental culture may contribute to emotions if residents feel that the focus is on performance and assessment rather than that of learning and improvement ^22^. Burnout among residents is an issue, with one study reporting rates of 42% among second-year residents ^23^. Emotional exhaustion is one of three dimensions of burnout ^24^, and the risk is increased when significant job responsibility is coupled with low autonomy ^25^. Although one goal of CBME is increased learner autonomy through enhanced self-reflection skills and self-directed learning, CBME residents bear increased responsibility for obtaining assessments. They have little autonomy, however, in terms of ensuring assessments are completed, or with the CBME transition. This additional assessment burden, coupled with the already-significant training demands, may increase their risk of burnout.

Research should examine CBME post-implementation, identifying resident reactions, and required support. Residents anticipate and experience benefits from CBME, but have both theoretical and practical concerns, which add emotional and administrative stress. It is important that training programs be aware of resident apprehensions regarding CBME, and seek to proactively address these through open, two-way communication before, during, and after implementation. This may help to alleviate concerns and uncertainty, and increase resident engagement and co-production which are instrumental to successful implementation ^9, 26^. Additionally, program leaders should acknowledge the potential for resident burnout, which may be exacerbated by the stress surrounding CBME implementation, and apply strategies to prevent, identify, and deal with added stress.

### Limitations

The sampling frame is unknown given that authors were dependent upon institutional PGME offices to recruit participants. Demographic data were not collected to maintain anonymity. The respondents represent a sample of the resident population in Canada and results may not be generalizable across the population. Additionally, some residents may have had little experience with CBME. Further, participants had varied backgrounds and we were unable to elicit whether there was a shared baseline knowledge of CBME. Lastly, the use of a survey to provide deep exploration of qualitative themes must be acknowledged as a limitation. However, it is important to recognize that participants provided rich narrative responses.

## Conclusion

This exploratory study is the largest known examination of resident perspectives of CBME in Canada, and using mixed methods allowed for thematic exploration, supporting, and supplementing the quantitative data. Training programs vary and resident perspectives on CBME will depend on their individual and institutional experiences. However, widespread reporting of administrative stressors associated with CBME and emotional responses to its implementation suggest that programs must be acutely aware of the risk of resident burnout and take proactive steps to address it.

## List of abbreviations

CBME      Competency-based medical education

PGME      Post-graduate medical education

RCPSC     Royal College of Physicians and Surgeons of Canada

## Data Availability

The data has not been made publicly available due to our institutional ethics clearance which outlines that only members of the research team would have access to the full raw and identifiable dataset. For those who are interested in accessing the data, please contact the corresponding author indicating your interest and outlining your reasons for access. The corresponding author will then review the institutional ethics clearance and identify next steps. The additional sample quotations provided are available for those who would like to review more of the data. Mendeley Data: CBME Resident Survey. https://doi.org/10.17632/xtr9dbt56d.1 ^16^. This project contains the following extended data: CBME_Environment_Final Survey.docx. (The file provides the final survey used to collect the data described in this study). Data are available under the terms of the Creative Commons Attribution 4.0 International license (CC-BY 4.0).
