# Supplementary material for: Exploring residents’ perceptions of competency-based medical education across Canada: A national survey study
Source: MedEdPublish (2016). 2024 Jan 8;14:2. [Version 1] doi: 10.12688/mep.19247.1 (PMC10933567; doi:10.12688/mep.19247.1)
Supplement: Peer review history

# Reviewer report: Reviewer response for version 1

**DOI:** 10.21956/mep.20620.r36182

**Published:** 2024-04-08

**Authors:** Jackson Hearn

**Affiliations:** 1 University of Cincinnati, Cincinnati, Ohio, USA

Dear Authors,  

 Thank you for the opportunity to review your recent article, “Exploring residents’ perceptions of competency-based medical education across Canada: A national survey study,” which approaches a meaningful topic to a broad range of medical educators and residents. Overall, the topic of resident perceptions of CBME (Competency Based Medical Education) is relevant to programs implementing CBME.  

 ---

 Summary: 

 This article presents data from a survey of 375 residents regarding perceptions of CBME (Competency Based Medical Education). The authors use closed ended (Likert scale) and open ended (typed narrative) responses. The respondents were grouped into either “pre-CBME” or “in CBME” groups and responses were compared using a mean difference of Likert items. Mann-Whitney U was used as a non-parametric comparison test. The authors conducted a thematic analysis from narrative responses. They identify the themes of “program outcome concerns, changes, and emotional responses,” and present representative quotes from narrative responses.  

 Strengths:  

 The sample is large and provides information describing participants' perceptions on various aspects of CBME. The authors’ qualitative analysis should be appreciated for its attention to the perspectives of residents who are (and will be) directly affected by implementation of CBME in training. Carefully evaluating resident perspectives is a crucial element in evaluating the quality of various elements of CBME implementation. The authors provide their survey instrument for review and ask about a number of important concerns regarding CBME, such as faculty interactions. The authors present compelling and illuminating quotations from residents. Their study improves our understanding of resident perspectives outside of resident responses to survey items. The quotes provided from the responses are compelling and engaging and support the codes and themes they propose. One particularly interesting idea which is found in several supporting quotes is the concern for ineffective implementation of CBME or persistent challenges which exist regardless of CBME (e.g. “the staff there don’t really care,” “there is a negative outlook on assessment). I think this offers a compelling area for further exploration. 

 Areas for Improvement: 

 The authors sampled residents from 10 Canadian institutions. The confidentiality provided by eschewing demographic data limits the degree to which sampling methods can be evaluated. For instance, the number of respondents from each institution is not given, which may obscure a pattern of low response rates at various institutions, or a pattern in which institutions provided respondents (e.g. urban vs rural, many vs few residents). While collecting demographic data may enable further insights (through methods such as subgroup analysis), this must be balanced against the utility of maintaining confidentiality for the participants. In other words, we do not know a lot about the participants, but that is precisely the point of confidentiality!  

 Regarding the quantitative analysis:  

 First, I would suggest that the use of the mean to represent central tendency for interval data (such as Likert *scale *responses) is controversial [1]. While Likert scales (consisting of multiple Likert items) have been considered interval data, individual Likert *items *remain ordinal data and must be analyzed as such. “Furthermore, because the numbers derived from Likert scales represent ordinal responses, presentation of a mean to the 100th decimal place is usually not helpful or enlightening to readers.”[2]. The authors use means of individual items in their analysis of survey results but do not display frequency distributions or report percentages of responses to compare response patterns for single Likert items. The items are not pooled into Likert scales in the analysis, which could allow the statistical comparisons the authors propose. Including measures of variability (or displaying data in a histogram or Likert chart) might better illuminate patterns among respondents. We might ask if participants favor extreme responses, or do they cluster around the mean? This could be improved by generating a histogram showing response frequencies or a Likert chart showing the proportions of responses at each level. In summary, I would encourage an review of the statistical methoda utilized with an experienced psychometrician to ensure the analytical methods are sound—I readily admit my training is not as a psychometrician, and the analyses conducted by the authors may be appropriate given particular features of this survey, study design, or data which are not in this version of the article.  

 Second, the authors utilize Cronbach’s alpha as a measure of reliability; however, normally distributed data are assumed when using this measure. The authors report that the sample violated normality assumptions. In addition, Cronbach’s alpha does not necessarily establish the reliability or internal consistency of responses when measuring different factors (such as faculty time constraints or definitions of competence). This could be improved by modifying the survey methodology to generate scales for multiple factors.  

 Regarding qualitative methods:

 Providing the codebook would enable more engagement with the qualitative analysis performed. This could be included in an appendix, as the survey instrument is. Theoretically, the authors hint at a relationship between the three themes (“Residents anticipate and experience benefits from CBME, but have both theoretical and practical concerns, which add emotional and administrative stress”); or to say another way, that concerns about CBME drive emotional and administrative stress. The quotes in support of theme 3 may better support the idea simply that residents experience stress more specifically related to tension between ensuring completion of CBME documentation and competing clinical workload (rather than concerns about CBME more generally). 

 Overall, I thank the authors for their diligent attention to gathering a wide range of perspectives from hundreds of residents and thoughtfully analyzing narrative responses on the topic. In the world of medical education, the importance of considering CBME from the viewpoint of residents cannot be understated.

Have any limitations of the research been acknowledged?

Yes

Is the work clearly and accurately presented and does it cite the current literature?

Yes

If applicable, is the statistical analysis and its interpretation appropriate?

Partly

Are all the source data underlying the results available to ensure full reproducibility?

No

Is the study design appropriate and is the work technically sound?

Yes

Are the conclusions drawn adequately supported by the results?

Partly

Are sufficient details of methods and analysis provided to allow replication by others?

Partly

Reviewer Expertise:

I study teaching communication to residents, resident assessment, and clinical competency committees.

I confirm that I have read this submission and believe that I have an appropriate level of expertise to confirm that it is of an acceptable scientific standard, however I have significant reservations, as outlined above.

## References

[1] : Resolving the 50-year debate around using and misusing Likert scales. *Med Educ* .2008;42(12) : 10.1111/j.1365-2923.2008.03172.x 1150-2 10.1111/j.1365-2923.2008.03172.x 19120943

[2] : Analyzing and interpreting data from likert-type scales. *J Grad Med Educ* .2013;5(4) : 10.4300/JGME-5-4-18 541-2 10.4300/JGME-5-4-18 24454995 PMC3886444

# Reviewer report: Reviewer response for version 1

**DOI:** 10.21956/mep.20620.r36042

**Published:** 2024-03-12

**Authors:** Ciraj Ali Mohammed

**Affiliations:** 1 Medical Education, College of Medicine and Health Sciences, National University of Science and Technology, Muscat, Oman

This study investigates Canadian residents' perceptions of competency-based medical education (CBME) highlighting areas of strength, and improvement within the CBME framework. The work contributes to a culture of continuous improvement in medical education, fostering transparency, accountability, and resident satisfaction in the field of CBME.

 Research of this nature can guide medical educators in refining curriculum design, assessment methods, and assist administrators in enhancing support structures that better align with the needs and expectations of residents

 Here are a few points that may be considered which help researchers in enhancing the robustness of the study, and provide a more comprehensive understanding of residents' experiences within the CBME framework.

**Sampling:**  You mention that a total of 10 Canadian institutions circulated the survey. What was the basis of selection for these institutes? 

 How did you ensure a representative sample of residents across various specialties and training levels? What was the method employed?

**Data collection: **The qualitative component appears limited, hindering a deep exploration of residents' lived experiences. A FGD or structured interview conducted with selected residents would have shed more light on the nature of concerns and sought ways of mitigation

**Analysis:**

 Did you have a provision to conduct subgroup analyses based on demographic factors (e.g., specialty, training level) to identify patterns and variations in perceptions? This may help in avoiding any unnecessary generalization of the nature of responses.

 How did you address confounding variables such as individual characteristics, personal experiences, or concurrent life events that would have influenced residents' perceptions independently of CBME?

**Discussion:**

 In light of your findings briefly suggest in the discussion: 

 a) Wellbeing measures proposed to alleviate stress and promote wellbeing among residents 

 b) Faculty development  measures that will be useful in the implementation of the CBME approach

 c) Plans for including residents' voices in the decision-making process

**Annexures:**

 Include the consensus-built codebook used in the study 

**Reviewer's Recommendations:** By providing evidence-based recommendations, the research serves as a valuable resource for institutions, policymakers, and educators striving to optimize CBME across the globe and hence can be accepted with possible inclusion/revision of suggested data/evidence.

Have any limitations of the research been acknowledged?

Yes

Is the work clearly and accurately presented and does it cite the current literature?

Yes

If applicable, is the statistical analysis and its interpretation appropriate?

Yes

Are all the source data underlying the results available to ensure full reproducibility?

Partly

Is the study design appropriate and is the work technically sound?

Yes

Are the conclusions drawn adequately supported by the results?

Yes

Are sufficient details of methods and analysis provided to allow replication by others?

Partly

Reviewer Expertise:

I have two decades of experience in Health Professions education with a special focus on active learning, PBL, faculty development, CBME, assessment, and accreditation.  I have served as a change management expert specifically in implementing educational intervention.

I confirm that I have read this submission and believe that I have an appropriate level of expertise to confirm that it is of an acceptable scientific standard.

# Reviewer report: Reviewer response for version 1

**DOI:** 10.21956/mep.20620.r36045

**Published:** 2024-03-06

**Authors:** Robert Cooney

**Affiliations:** 1 Center for Faculty and Professional Development, Geisinger Medical Center, Danville, Pennsylvania, USA

Dear Authors,

 I have reviewed your article "Exploring residents' perceptions of competency-based medical education across Canada: A national survey study" [version 1; peer review: awaiting peer review] and overall I find it to be a well-conducted study that provides important insights into how Canadian residents view competency-based medical education (CBME). I believe the article has academic merit and will be an important contribution to the literature on this topic. However, I do have some suggestions for improvement, which I outline below.

 Summary:

 The study collected survey data from 375 medical residents across Canada to examine their perceptions and experiences with the transition to CBME. The quantitative data was analyzed appropriately and the qualitative data from open-ended responses yielded rich insights through thematic analysis. Three main themes emerged: program outcome concerns, changes resulting from CBME, and emotional responses to CBME.  

 Strengths:

 - Large national sample across multiple programs and specialties

 - Mixed-methods design allowing for exploration of quantitative trends as well as deeper insights from qualitative data

 - Transparent reporting of methods, analysis, and researcher reflexivity 

 - Findings align with and expand on existing literature regarding resident perceptions of CBME

 - Highlights important areas for programs to focus on, such as addressing logistical challenges, faculty buy-in, and resident stress/burnout risk

 Areas for Improvement:

 1. Reproducibility and Details for Replication 

 While the methods are described thoroughly overall, replication will be difficult

 - The survey instrument is provided, as an appendix, which is a helpful first step. I would like to see:

 - Details on the sampling approach and response rate are lacking, specifically:

    - How institutions/programs were selected for participation 

    - What was the total eligible population for each group (pre-CBME and CBME residents)

    - What were the response rates for each group relative to the eligible population (I acknowledge this is likely unknowable given the structure of how the survey was administered and the decision to not collect demographic data).

 Providing these additional sampling and response details is important for assessing potential selection bias and better understanding the representativeness of the sample. This would also help to know the total possible pool of respondents. 

 - More details on the qualitative coding process would be helpful. Again, the codebook could also be offered as a supplementary appendix and it would be helpful to know what theory guides the qualitative process (grounded theory, etc). Guessing this was a simple thematic analysis vs narrative analysis but it’s always helpful to spell this out in writing.

 Main Revisions Needed:

 To address the key points above and improve the scientific merit of the study, I would recommend the authors:

 1) Provide additional details on the sampling approach, population sizes, and response rates for context.

 2) Expand on the description of the qualitative coding process (consider supplemental appendix). Also, which type of qualitative theory informed your work?

 Other than those points, the article is very well-written and follows a clear structure. The literature review appropriately sets up the study's rationale and the limitations are discussed transparently. With the additional details and data transparency suggested above, I believe this article would make a strong contribution to the literature.

 I hope these comments are helpful for revising the manuscript.

Have any limitations of the research been acknowledged?

Yes

Is the work clearly and accurately presented and does it cite the current literature?

Yes

If applicable, is the statistical analysis and its interpretation appropriate?

Yes

Are all the source data underlying the results available to ensure full reproducibility?

Partly

Is the study design appropriate and is the work technically sound?

Yes

Are the conclusions drawn adequately supported by the results?

Yes

Are sufficient details of methods and analysis provided to allow replication by others?

Partly

Reviewer Expertise:

Medical Education; educational technology, health systems science, faculty development

I confirm that I have read this submission and believe that I have an appropriate level of expertise to confirm that it is of an acceptable scientific standard.
